# COVID-19 vaccination lowers SARS-CoV-2 infection risk independent of diabetes, cancer and smoking in SHIP-COVID cohort, Northern Germany

**DOI:** 10.1038/s41598-025-22334-2

**Published:** 2025-10-07

**Authors:** Muhammad Nasir Khan Khattak, Josefin Pauline Haß, Till Ittermann, Marcus Dörr, Ola Sidahmed, Niels Ole Kristiansen, Lena Ulm, Kathrin Lehmann, Karsten Becker, Tillman Görig, Nils-Olaf Hübner, Henry Völzke

**Affiliations:** 1 Department of SHIP/Clinical-Epidemiological Research, Institute for Community Medicine, University Medicine Greifswald, Walther Rathenau Str. 48, 17475 Greifswald, Germany; 2 Institute for Hygiene and Environmental Medicine, University Medicine Greifswald, Greifswald, Germany; 3 Institute of Molecular Biology, Mainz, Germany; 4 Friedrich-Loeffler-Institute of Medical Microbiology, University Medicine Greifswald, Greifswald, Germany

**Keywords:** SARS-CoV-2, COVID-19, Vaccination, Diabetes, Cancer, Smoking, Cancer, Diseases, Immunology, Medical research, Risk factors

## Abstract

This study (*n* = 668) investigated the effect of vaccination on SARS-CoV-2 infection among individuals with type 2 diabetes mellitus (T2DM), cancer, and smoking in a prospective cohort study in Northern Germany. Participants from the SHIP-COVID cohort were followed from October 2020 to October 2022 using repeated questionnaires and serological tests. Infection was defined by anti-nucleocapsid (anti-NCP) antibodies’ detection, self-reported Real Time Polymerase Chain Reaction (RT-PCR) or rapid antigen test. Vaccine efficacy was evaluated using risk differences (RD), risk ratios (RR), p-values, and 95% confidence intervals. Infection risk decreased with more vaccine doses; the 3rd (RR = 0.68) and 4th (RR = 0.29) doses offered significant protection. Both sexes showed protection with 3rd and 4th doses (RR = 0.67 and 0.20 in women and 0.68 and 0.39 in men, respectively). Logistic regression showed higher infection odds in individuals with T2DM (14%) and cancer (68% in SHIP-START-3 dataset and 45% in SHIP-START-4 dataset), while current smokers had 47% and 45% lower odds than non-smokers in SHIP-START-3 and SHIP-START-4 datasets respectively. No significant interactions were found between vaccination and T2DM, cancer, or smoking.

**Supplementary Information:**

The online version contains supplementary material available at 10.1038/s41598-025-22334-2.

## Introduction

Germany started its COVID-19 vaccination program in late December 2020. The early phase prioritized high-risk groups, including healthcare workers, elderly people, and individuals with underlying health conditions^1,2^. Since the first dose was administered on December 26th 2020, Germany has delivered over 193 million vaccine doses, with 78% of the population received at least one dose and 63% of the population received at least one booster^3^.

Available literature is reporting a complex bidirectional association of diabetes and COVID-19 vaccination. In individuals with type 2 diabetes mellitus (T2DM), the risk of glycaemia is reportedly increased and their immune response is lower after vaccination compared to general population, but can be increased with glycemic management^4,5^. Moreover, it was also reported that the incidence of diabetes is increased after a COVID-19 infection. Diabetes persisted in those who were hospitalized compared to those who were not, but less obvious in individuals vaccinated against COVID-19^6^. This finding is further supported by a large study showing a more protection with vaccination in individuals with T2DM compared to those without. This protection was even increased with booster doses^7^.

Cancer patients represent a high risk group of severe COVID-19 also because of their age, disease, treatment, and medical co-morbidities^8^. COVID-19 vaccination plays a crucial role in reducing the infection risk and severe outcomes in cancer patients, who are particularly vulnerable due to their immunocompromised status^9^. Further categorization can be done based on the type of cancer. Patients with hematologic cancers have higher risk of SARS-CoV-2 infection compared to patients with other cancer types or those without cancer. However, this risk of infection can be minimized using boosters with triple vaccination^10,11^.

Smoking is widely recognized to have negative health consequences, but its relationship with COVID-19 remains a topic of debate^12^. A recently published cross-sectional study suggests a potential association between smoking and a reduced risk of SARS-CoV-2 infection^13^. Similar findings have been reported in other studies^14–16^ while several others contradict this protective effect of smoking against SARS-CoV-2 infection^17–19^.

Individuals with T2DM and cancer are considered immunocompromised due to impairment in innate and adaptive immune response in diabetics^20^, and immunosuppression due to multiple cancer related factors in cancer patients^21^. As a result, these individuals are considered as high-risk groups for SARS-CoV-2 infection. Additionally, smoking is recognized as a significant risk for pulmonary diseases, including COVID-19. In this context, our study aimed to evaluate the protective effects of different vaccination doses in the general population of North Germany. Furthermore, we sought to investigate the association of T2DM, cancer and smoking with SARS-CoV-2 infection in diversely vaccinated individuals of the SHIP-COVID cohort in Northern Germany.

## Methods

### Study population

**Fig. 1:**
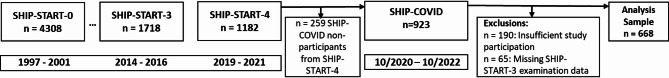
Schematic presentation of the study population.

We performed analyses using data from the Study of Health in Pomerania (SHIP), which was and is being conducted in Northeast Germany with several cohorts. Initial recruitment for the SHIP-START cohort took place from 1997 until 2001 with a total of 4308 participants (response 68.8%). We used data from the third follow-up of SHIP-START (SHIP-START-3), where 1718 individuals (response 54.7% in still living participants) aged 41–89 years were examined between 2014 and 2016. The fourth follow up i.e. SHIP-START-4 with 1182 participants (response 44.5% in still living participants) were examined during 2019 to 2021^22^. At the advent of COVID-19 pandemic in March 2020, SHIP started recruitment for the SHIP-COVID study from SHIP-START-4 participants. A total of 923 individuals from the SHIP-START-4 population were recruited for SHIP-COVID. Of these 923 individuals, 590 already finished their SHIP-START-4 examination (Subgroup 2 – SG2). For the other 333 individuals, the SHIP-START-4 examination was still pending (Subgroup 1 – SG1). Participants were followed up on, from October 2020 until October 2022 on a monthly basis for SG1 and a 4 monthly basis for SG2. We pooled the groups for this study. From these 923, we chose only those for further analysis who sent ≥ 11 follow-up questionnaires in SG1 and ≥ 3 follow-up questionnaires in SG2. This left us with a total of 733 participants for SHIP-START-4. Of these 733, only 668 participants fulfilled the inclusion criteria for SHIP-START-3 (Fig. 1). We used SHIP-START-3 for this study, as participants’ T2DM, cancer, and smoking status were well established before the COVID-19 pandemic. As a robustness check, we also included SHIP-START-4 data for cancer and current smoking, to evaluate whether any changes since SHIP-START-3 influenced the results. Age of the participants was considered at the time of the recruitment for the SHIP-COVID (range 42–90 years). The participants were asked to fill the questionnaire and send their dry blood samples on a monthly basis for SG1 and 4 monthly bases for SG2. Therefore, we analyzed the association of COVID-19 infection status with underlying conditions based on SHIP-START-3 examinations from 668 participants who regularly submitted questionnaires and blood samples at the described intervals. Details of the genomic epidemiology of SARS-CoV-2 variants circulating in the sera during this period are described elsewhere^23^.

The study was conducted according to the guidelines laid down in the Declaration of Helsinki, and the protocol was approved by the Ethical Review Board of the University Medicine Greifswald (BB118/20). All subjects gave their informed consent to be included in the study.

### Vaccination

The participants were vaccinated as a part of the national vaccination campaign, which continued throughout the study period. A large number of participants (92.8%; 91.1% of males and 94.15% females) reported to be vaccinated at least once. The commonly used mRNA-based vaccines (Comirnaty from Pfizer-BioNtech, Elasomeran from Moderna) and non-mRNA vaccines (AstraZeneca, Johnson and Johnson and Novavax) were developed to encode the spike protein of SARS-CoV-2, thereby triggering the production of specific anti-spike antibodies. For our study we counted only those vaccines that were administered before any infection.

### Prevalence

As mentioned above, the vaccines used are leading to generation of anti-spike antibodies. The presence of anti-nucleocapsid (anti-NCP) protein antibodies indicates prior exposure to the SARS-CoV-2 virus, as the nucleocapsid protein is produced only during natural infection and is not included in the currently available vaccines^24,25^. Therefore, this information was used to identify infected cases for the present analysis in addition to the self-reported infection by real time polymerase chain reaction (RT-PCR) or rapid antigen tests.

The tests for anti-SARS-CoV-2 antibodies (both anti-spike and anti-NCP) were carried out using assay from Euroimmun (Anti-SARS-CoV-2- ELISA (IgG and Anti-SARS-CoV-2-NCP-ELISA (iGG); Euroimmun, Lübeck, Germany). Since these assays were validated for both serum and dried blood samples (DBS)^26^, initial and follow-up examinations could be conducted within the same testing system. Testing for IgG antibodies against the spike SI-domain and nucleocapsid protein was performed in accordance with the manufacturer’s instructions at the Friedrich-Loeffler-Institute for Medical Microbiology at the University medicine Greifswald (UMG). At baseline, venous and capillary blood were tested in parallel for quality assurance. In the DBS test (Euroimmun), self-sampling was reduced to a short prick of the fingertip and was therefore a minimally invasive blood sample. The test kits were dispatched by standard post for participants to carry out at home and send back after usage. Residual serum samples were stored at − 80 °C in the biobank at Institute for Clinical Chemistry and Laboratory Medicine at the UMG for future analyses.

Participants were classified as infected if they exhibited an Anti-NCP antibody ratio of > 1.1 at least once or reported a positive result from either RT-PCR or rapid antigen testing.

### Underlying conditions

In SHIP, participants are tested for serum glucose and HbA1c and asked for reported diabetes. Participants who self-reported T2DM or HbA1c ≥ 6.5% were defined as positive. Among the 59 participants with T2DM, 37 were receiving antidiabetic medication. Positivity for cancer (none was receiving anti-cancer medication), stroke, and myocardial infarction was based on self-reported data collected via questionnaire. We excluded stroke and myocardial infarction from the analysis due to the low number of positive participants (Table 1).

### Behavioral factors

As part of the SHIP-examinations, smoking status was assessed via computer assisted personnel interviews. This includes (among others) questions regarding current smoking status, smoking intensity (number of consumed cigarettes), and whether the individual previously smoked cigarettes and when they started. We used current smoking as a variable for our analysis.

### Statistical analyses

To estimate the protective effect of vaccination we calculated risk difference (RD) and risk ratios (RR) with “no vaccination” as a reference. We also calculated RD and RR between males and female participants. Additionally, we repeated these calculations stratified by sex.

Adjusted binary logistic regression was used to evaluate the association of having a SARS-CoV-2 infection with T2DM, cancer and smoking. SARS-CoV-2 infection was an outcome variable, and diabetes, cancer and smoking were used as predictor variable separately. To account for potential confounding effects of the number of vaccinations, sex and age on the prediction, we adjusted the model for these variables. Furthermore, we also measured the interaction of T2DM, cancer and smoking with the number of vaccinations. A sample equation is given below.

$$\log (P({\text{infection}} = {\text{Yes}})/{\text{P}}({\text{infection}} = {\text{No}})) = \beta 0 + \beta 1({\text{predictor}}) + \beta 2({\text{number}}\;{\text{of}}\;{\text{vaccinations}}) + \beta 3({\text{Sex}}) + \beta 4({\text{age)}}$$

We used odds ratios (OR) and 95% confidence intervals (CI) to assess the strength and significance of the association. A p level of < 0.05 was considered statistically significant. Analyses were conducted using R version 4.1.1.

## Results

The characteristics of the study population are shown in Table 1 stratified by SARS-CoV-2 infection status and sex. The study population included 668 individuals, of whom 335 (50.15%) were infected at least once throughout the study period. Women accounted for 192 (57.31%) of the infected participants and 184 (55.26%) of the not-infected participants. The mean age of infected women and men was 59.3 ± 10.7 and 62.7 ± 11.5 years, respectively. Among not-infected participants, women had a mean age of 64.8 ± 10.7 years and men of 65.8 ± 11.1years. Among the infected individuals, 56 (including 27 women) were never vaccinated throughout the study period, 6 (4 women) were vaccinated once, 33 (21 women) were vaccinated twice, 219 (133 women) were vaccinated thrice, and 21 (7 women) were vaccinated four times. Among not-infected individuals, 19 (8 women) were never vaccinated throughout the study period, 6 (2 women) were vaccinated once, 22 (11 women) were vaccinated twice, 212 (124 women) were vaccinated thrice, and 74 (39 women) were vaccinated four times. With respect to underlying conditions, 59 had T2DM, 25 had cancer in SHIP-START-3 dataset and 74 in SHIP-START-4 dataset, 16 had myocardial infarction and 13 had stroke. Regarding smoking, 114 were current smokers in SHIP-START-3 dataset and 93 in SHIP-START-4 dataset (Table 1).

**Table 1:** Summary table of study population by infection status and sex.

Variable	Infected (*n* = 335, 50.15%)		Not infected (*n* = 333, 49.85%)	
Demographic parameters				
Sex	Women (*n* = 192, 57.31%)	Men (*n* = 143, 42.69%)	Women (*n* = 184, 55.26%)	Men (*n* = 149, 44.74%)
Age (mean ± SD)	59.3 ± 10.7	62.7 ± 11.5	64.8 ± 10.7	65.8 ± 11.1
Underlying conditions				
Type 2 diabetes mellitus (T2DM)	13 (6.77%)	13 (9.10%)	19 (10.33%)	14 (9.40%)
Cancer SHIP-START-3	7 (3.65%)	6 (4.20%)	6 (3.26%)	6 (4.03%)
Cancer SHIP-START-4	18 (9.38%)	20 (13.99%)	20 (10.87%)	16 (10.74%)
Myocardial infarction	0 (0.00%)	6 (4.20%)	3 (1.63)	7 (4.70%)
Stroke	3 (1.56%)	4 (2.80%)	2 (1.10%)	4 (2.68%)
Behavioral parameter				
Current smoker SHIP-START-3	37 (19.27%)	13 (9.10%)	35 (19.02%)	29 (19.46%)
Current smoker SHIP-START-4	33 (17.19%)	8 (5.59%)	27 (14.67%)	25 (16.78%)
Vaccination				
0-vaccine	27 (14.06%)	29 (20.28%)	8 (4.35%)	11 (7.38%)
1-vaccine	4 (2.08%)	2 (1.40%)	2 (1.09%)	4 (2.68%)
2- vaccines	21 (10.94%)	12 (8.39%)	11 (5.98%)	11 (7.38%)
3-vaccines	133 (69.27%)	86 (60.14%)	124 (67.39%)	88 (59.06%)
4-vaccines	7 (3.65%)	14 (9.79%)	39 (21.20%)	35 (23.49%)
Total	192	143	184	149

### Protective effects of vaccination

The results for RD and RR by number the of vaccinations can be found in Table 2. In vaccinated participants, the risk of SARS-CoV-2 infection showed a dose dependent reduction compared to the unvaccinated participants (75%). However, an exception was observed after the first dose, where the reduction was 50% followed by a slight increase after two doses (60%). The risk then decreased again with three doses (51%) and further with four doses (20%). This fluctuation may be explained by a small number of participants in the single dose group. In addition, one fourth of the participants in a single dose group received Johnson and Johnson vaccines, where single dose is considered as complete vaccination.

Similar pattern was observed for risk difference. The risk difference indicates a greater protective effect in vaccinated individuals, with increasing protection with increasing doses of vaccinations (-0.25, − 0.15, − 0.24, and − 0.53 for one to four vaccinations, respectively), compared to unvaccinated individuals.

The risk ratios for vaccinated individuals compared to unvaccinated individuals also declined (0.67, 0.8, 0.68, and 0.29 for one to four vaccinations, respectively) with increasing doses of vaccinations. However, statistically significant protection was observed with three and four doses only (Table 2).

**Table 2:** Estimated risk, risk difference (RD), risk ratio (RR) with 95% confidence interval (CI) and p values by vaccination status.

Vaccination status	Infected (*n*)	Not Infected (*n*)	Total	Risk	RD	RR (CI 95%)	*p*-value
0	56	19	75	0.75	Reference	Reference	
1	6	6	12	0.50	− 0.25	0.67 (− 0.55 to 0.05)	0.08
2	33	22	55	0.60	− 0.15	0.8 (− 0.31 to 0.02)	0.08
3	219	212	431	0.51	− 0.24	0.68 (− 0.35 to 0.13)	0.00
4	21	74	95	0.22	− 0.53	0.29 (− 0.65 to 0.4)	0.00
Total	335	333	668				

We also analyzed the risk difference for men and women; however, we didn’t find a significant difference (RD = 0.02 and RR = 0.96 (CI 95%= 0.82–1.12) (Table 3).

**Table 3:** Risk difference (RD) and risk ratio (RR) by sex.

	Infected (*n*)	Not Infected (*n*)	Total	Risk	RD	RR (CI 95%)	*p*-value
Men	143	149	292	0.49	Reference	Reference	
Women	192	184	376	0.51	0.02	0.96 (0.82–1.12)	0.65
Total	335	333	668				

We also analyzed the protective effect of vaccination separately in men and women. We observed similar trends of decreasing RD and RR in both sexes as the number of vaccinations increased. Significant protection was achieved with three and four doses of the vaccines in both groups (Table 4).

**Table 4:** Risk difference (RD) and risk ratio (RR) by vaccination in women and men.

No of vaccination	Infected (*n*)	Not Infected (*n*)	total	Risk	RD	RR (CI 95%)	*p*-value
(a) Risk difference (RD) and risk ratio (RR) by vaccination in women							
0	27	8	35	0.77	Reference	Reference	
1	4	2	6	0.67	− 0.11	0.86(0.48–1.57)	0.62
2	21	11	32	0.66	− 0.12	0.85(0.63–1.16)	0.42
3	133	124	257	0.52	− 0.25	0.67(0.54–0.83)	0.01
4	7	39	46	0.15	− 0.62	0.20(0.10–0.40)	0.00
Total	192	184	376				
(b) Risk difference (RD) and risk ratio (RR) by vaccination in men							
0	29	11	40	0.73	Reference	Reference	
1	2	4	6	0.33	− 0.39	0.46 (0.15–1.45)	0.08
2	12	11	23	0.52	− 0.203	0.72(0.47–1.11)	0.17
3	86	88	174	0.49	− 0.231	0.68(0.54–0.87)	0.01
4	14	35	49	0.29	− 0.44	0.39(0.24–0.64)	0.00
Total	143	149	292				

We further analyzed age as a separate predictor of the SARS-CoV-2 infection and that increasing age was significantly associated with greater protection (OR = 0.96, 95% CI = 0.95–0.98).

### Association of SARS-CoV-2 infection with T2DM, cancer and smoking

We used adjusted binary logistic regression to analyze the association of T2DM, cancer and smoking with the SARS-CoV-2 infection adjusted for number of vaccinations, sex and age.

A 14% increase in infection was observed among individuals with T2DM (OR  1.14, 95% CI 0.64–2.04; *p* = 0.66). Although this association did not reach statistical significance, the OR indicates a potential positive trend. Furthermore, we also analyzed the interaction of T2DM with vaccinations, and found no significant interaction (Tables S1 and S2).

For individuals with cancer as an underlying condition, the infection rate was 68% higher using SHIP-START-3 data and 45% higher using SHIP-START-4 data compared to those without cancer. However, this increase in both cases was not statistically significant (SHIP-START-3: OR  1.68, *p* = 0.25, 95%CI  0.69–4.14, SHIP-START-4: OR  1.45, *p* = 0.17, 95% CI  0.86–2.46), though the OR suggests a positive trend in both datasets. This might also be attributed to a low number of participants. After analyzing the interaction of cancer with vaccinations, we found no significant interactions (Tables S1 and S2).

We found an inverse association (*p* < 0.05) between smoking and SARS-CoV-2 infection ((SHIP-START-3: OR  0.53, *p* = 0.00, 95%CI  0.34–0.83, SHIP-START-4: OR  0.55, *p* = 0.01, 95% CI  0.34–0.88). After analyzing the interactions of smoking status with vaccinations, we found no significant interactions of smoking status with vaccination in prediction of SARS-CoV-2 infection (Tables S1 and S2).

## Discussion

Similar to other vaccines, COVID-19 vaccination saved millions of lives during the COVID-19 pandemic by reducing the incidence of severe COVID-19 cases^27^. The findings of this study suggest a strong inverse relationship between the number of vaccine doses and the risk of infection. Individuals who received no vaccination had the highest infection rate (75%), while those with increasing doses showed a progressive decline in risk. Notably, three and four doses provided significant protection, with infection rates dropping to 51% and 22%, respectively. The relative risk (RR) for three and four doses was 0.68 and 0.29, indicating a 32% and 71% reduction in infection risk compared to the unvaccinated group. These results were statistically significant (*p* < 0.001), with confidence intervals that did not include 1, confirming the protective effect of vaccination. However, one and two doses showed only a moderate decrease in infection risk, and their confidence intervals included 1, suggesting no statistically significant protection at lower doses. These findings highlight the importance of receiving a full vaccination regimen, as incomplete vaccination may not provide sufficient immunity. The generalizability of these findings to other age groups is uncertain, as our dataset included participants aged 42–90 years. However, this age range is particularly relevant, as they are considered more vulnerable to SARS-CoV-2 infection. Further investigation into immune response dynamics and booster effects is warranted to optimize vaccination strategies for infection control.

Our findings are supported by a large observational study, which demonstrated that a third dose of the COVID-19 vaccine is more effective in preventing severe outcomes compared to two doses^28^. Similarly, a study focusing on individuals aged 60 and above found that those who received three doses experienced lower infection rates and fewer severe outcomes than those who had only two doses^29^. Notably, booster doses have been shown to offer greater protection not only compared to receiving two doses of the vaccine but also compared to immunity gained from a prior infection^30^. The vaccine effectiveness of a third or booster dose has been reviewed recently where the effectiveness of a third dose against the completed vaccination group (i.e. individuals who received two doses) was higher in terms of infection and its severe outcomes including hospitalization and death. However, it was not same for all variants of the virus^31,32^. The study period spanned two complete waves (Alpha and Delta) and the onset of the third wave (Omicron) of SARS-CoV-2 variants of concerns (VOCs). These three VOCs appeared with a delay of several weeks in Mecklenburg-Western Pomerania, the Germany federal state in whose north-eastern region the study took place^23^.

Patients with T2DM are generally more susceptible to bacterial and viral infections compared to the general population^33^. Few earlier studies during the initial phase of the COVID-19 pandemic, before widespread vaccination, reported elevated infection risk for COVID-19 and more severe outcomes in T2DM patients compared to the non-T2DM individuals from different regions of the world^34–37^. Our study conducted between October 2020 and October 2022 – a period overlapping the rollout of COVID-19 vaccination – also found 14% higher risk and statistically not significant (*p* > 0.05) of SARS-CoV-2 infection in participants with T2DM compared to those without T2DM. Our findings indicate that T2DM slightly increases the likelihood of SARS-CoV-2 infection within our study population despite the high vaccination coverage of the participants. A recent mathematical model demonstrated that higher vaccination doses significantly reduce infection rates in T2DM patients, highlighting the critical role of COVID-19 vaccination in this vulnerable group. Vaccination not only mitigates infection risks but also substantially decreases the severity of disease outcomes, further highlighting its importance in improving overall health outcomes for individuals with diabetes^38^. Considering this modelling we speculate that the T2DM participants could have higher infection rate if left unvaccinated.

Similarly, a large retrospective study in Spain also found that individuals with T2DM who received a third booster dose were significantly better protected (84% with booster dose vs. 65% with no booster dose) against SARS-CoV-2 infection and severe outcomes^39^. It is important to recognize that individuals with diabetes are more vulnerable to infections at baseline due to their weaker or compromised immune response. However, COVID-19 vaccination, particularly when continued through booster doses, provides significant protective benefits for diabetics. Despite all the heterogeneity of the studies, it is established that vaccination is highly protective against COVID-19 and no difference is recorded in mortality between vaccinated individuals with and without T2DM^40^. Further supporting these findings are studies conducted on seroconversion rates in people with and without T2DM after vaccination. Many studies found no statistical difference in seroconversion rates between individuals with and without T2DM after COVID-19 vaccination^41–43^. One large study however reported lower seroconversion rates in people with diabetes after first dose but this disparity was resolved following the second dose, where seroconversion rates reached 100% with detectable anti-spike antibodies^44^.

To further investigate the influence of diabetes, we examined its interaction with three- and four-doses of COVID-19 vaccination status within the regression model. However, these interaction analyses also failed to reveal significant associations. On the one hand, these findings suggest that while diabetes is a well-recognized risk factor for SARS-CoV-2 infection and also severe outcomes, it might not significantly influence susceptibility to infection when controlling for key demographic and vaccination variables. On the other hand, our study population may have been too small to detect statistically significant interactions.

As an accepted phenomenon, cancer patients are immunocompromised and are therefore considered at higher risk of catching a SARS-CoV-2 infection compared to the general population. However, studies regarding vaccine efficacy using SARS-CoV-2 infection prevalence as an outcome are limited. Most of the studies have used seroconversion as an outcome and have reported poor immune response with a single dose of mRNA vaccine in cancer patients. However, it was enhanced to above 90% of the patients after the 2nd dose, but differences in immune titers remained based on e.g. type of tumor and treatment stage^45–48^.

Our study evaluated the relationship between cancer as an underlying condition and the risk of SARS-CoV-2 infection. The analysis revealed no statistically significant association suggesting that cancer may not independently predispose individuals to a higher likelihood of SARS-CoV-2 infection in our cohort. However, the observed odds ratios (OR 1.68 and 1.45 Tables S1 and S2) points to a possible trend toward an association that did not reach statistical significance, potentially due to vaccination of the population. A large retrospective study conducted in Spain reported that cancer patients who received a third booster dose exhibited substantially greater protection against both SARS-CoV-2 infection and severe outcomes, with effectiveness rates of 81% compared to 54% in those who did not receive the booster^39^.

To further explore this trend, we analyzed the interaction between cancer and vaccination status, specifically among individuals receiving three or four doses of COVID-19 vaccines. The results demonstrated no significant interaction between cancer and vaccination status in reducing the risk of SARS-CoV-2 infection. These findings are depicted in Table S1, reinforcing the lack of a definitive association between cancer and SARS-CoV-2 infection risk when accounting for vaccination status. However, the low study population might also be a reason for these findings.

Association of smoking and SARS-CoV-2 infection is debatable because of the counterintuitive findings. Smoking is a well-established risk factor for infectious respiratory diseases, morbidity and mortality^49–51^. However, our findings indicate statistically significant negative association between smoking and SARS-CoV-2 infection. This counterintuitive finding needs very cautious discussion and interpretation and must not be used to encourage cigarette smoking. Although several findings and opinions support the expected negative and severe consequences of COVID-19 in smokers compared to non-smokers but doesn’t investigate infection risk^52^ and our results do not contradict them. Rather our results (OR 0.53 and 0.55 Table S1 and S2) are concordant with a meta-analysis of 13 studies across all age groups suggesting a reduced risk ratio (RR = 0.74) of infection in current smokers compared to never smokers^53^. Another study in Korea reported that, among participants older than 40 years, had a lower risk of SARS-CoV-2 infection in current smokers (OR 0.33, 95% CI 0.28–0.38) compared to former smokers (OR 0.81, 95% CI 0.78–0.91) and never smokers^54^. Similarly, data from 38 European countries is also reporting the reduced risk of SARS-CoV-2 infection in smokers^15^. Some studies hypothesize that nicotine might downregulate angiotensin-converting enzyme 2 (ACE2) expression that serves as a receptor for the SARS-CoV-2^55^. However, this needs further investigation in order to understand the mechanism behind any protective role of nicotine. Whether deviant behavior patterns of smokers have also contributed to this phenomenon is completely unclear. We also investigated interaction of smoking with vaccination, however did not find any significant interaction.

Our study concludes that vaccination remains a critical player in mitigating SARS-CoV-2 infection risk regardless of diabetes, cancer and smoking status. The lack of interaction between vaccination and diabetes, cancer and smoking are suggestive of vaccination benefits across all conditions and behavioral subgroups. However, in diabetes and cancer, we suggest that booster doses provide substantial protective benefits.

Limitations of our study include the lack of baseline data prior to the start of vaccination, as participants could not be reached due to lockdowns and other complications. Additionally, the sample size is limited for participants with T2DM and cancer and smokers. Finally, there is a chance that individuals developed T2DM after their third examination but before the start of the pandemic. Therefore, we cannot rule out that some individuals are misclassified as not diabetic even though they are.

## Supplementary Information

Below is the link to the electronic supplementary material.

Supplementary Material 1

## Data Availability

The datasets generated and/or analyzed during the current study are not publicly available due to restrictions imposed by the Data Protection Act but are available from the corresponding author on reasonable request. Access can be granted to qualified researcher after submission of a detailed proposal mentioning the scientific purpose of the data request and approval from the institution review board.
