# Supplementary material for: COVID-19 vaccination lowers SARS-CoV-2 infection risk independent of diabetes, cancer and smoking in SHIP-COVID cohort, Northern Germany
Source: Sci Rep. 2025 Oct 7;15:35032. doi: 10.1038/s41598-025-22334-2 (PMC12504655; doi:10.1038/s41598-025-22334-2)
Supplement: Supplementary file 1 — Supplementary Material 1 [file 41598_2025_22334_MOESM1_ESM.docx]

| Independent Variable | Type | Term | OR(95%CI) | p_value |
| --- | --- | --- | --- | --- |
| **T2DM** | **Main Effect** | **T2DM** | **1.14 (0.64–2.04)** | **0.66** |
| T2DM | Main Effect | Vaccine/s adminsitered1 | 0.41 (0.11–1.46) | 0.16 |
| T2DM | Main Effect | Vaccine/s adminsitered2 | 0.52 (0.24–1.11) | 0.09 |
| T2DM | Main Effect | Vaccine/s adminsitered3 | 0.38 (0.21–0.65) | 0.00 |
| T2DM | Main Effect | Vaccine/s adminsitered4 | 0.13 (0.06–0.27) | 0.00 |
| T2DM | Main Effect | Sex (men reference) | 1.04 (0.75–1.43) | 0.82 |
| T2DM | Main Effect | Age | 0.98 (0.96–0.99) | 0.01 |
| **Cancer** | **Main Effect** | **Cancer** | **1.68 (0.69–4.14)** | **0.25** |
| Cancer | Main Effect | Vaccine/s adminsitered1 | 0.42 (0.12–1.52) | 0.18 |
| Cancer | Main Effect | Vaccine/s adminsitered2 | 0.53 (0.25–1.13) | 0.10 |
| Cancer | Main Effect | Vaccine/s adminsitered3 | 0.39 (0.22–0.67) | 0.00 |
| Cancer | Main Effect | Vaccine/s adminsitered4 | 0.13 (0.06–0.27) | 0.00 |
| Cancer | Main Effect | Sex (men reference) | 1.04 (0.75–1.43) | 0.83 |
| Cancer | Main Effect | Age | 0.98 (0.96–0.99) | 0.01 |
| **smoking** | **Main Effect** | **smoking** | **0.53 (0.34–0.83)** | **0.00** |
| smoking | Main Effect | Vaccine/s adminsitered1 | 0.44 (0.12–1.59) | 0.20 |
| smoking | Main Effect | Vaccine/s adminsitered2 | 0.52 (0.24–1.11) | 0.09 |
| smoking | Main Effect | Vaccine/s adminsitered3 | 0.38 (0.21–0.66) | 0.00 |
| smoking | Main Effect | Vaccine/s adminsitered4 | 0.13 (0.06–0.27) | 0.00 |
| smoking | Main Effect | Sex (men reference) | 1.05 (0.76–1.46) | 0.75 |
| smoking | Main Effect | Age | 0.97 (0.96–0.99) | 0.00 |
| T2DM | Interaction | T2DM:Vaccine/s adminsitered1 | 2693764.37 (0–NA) | 0.98 |
| T2DM | Interaction | T2DM:Vaccine/s adminsitered2 | 0.88 (0.02–21.18) | 0.94 |
| T2DM | Interaction | T2DM:Vaccine/s adminsitered3 | 1.97 (0.2–19.52) | 0.54 |
| T2DM | Interaction | T2DM:Vaccine/s adminsitered4 | 10.43 (0.92–118.27) | 0.05 |
| Cancer | Interaction | Cancer:Vaccine/s adminsitered1 | NA (NA–NA) |  |
| Cancer | Interaction | Cancer:Vaccine/s adminsitered2 | 514844.29 (0–NA) | 0.98 |
| Cancer | Interaction | Cancer:Vaccine/s adminsitered3 | 0.39 (0.01–4.47) | 0.49 |
| Cancer | Interaction | Cancer:Vaccine/s adminsitered4 | 2.86 (0.11–32.94) | 0.43 |
| smoking | Interaction | smoking:Vaccine/s adminsitered1 | 0.55 (0.02–11.22) | 0.71 |
| smoking | Interaction | smoking:Vaccine/s adminsitered2 | 0.28 (0.03–1.91) | 0.21 |
| smoking | Interaction | smoking:Vaccine/s adminsitered3 | 0.92 (0.21–3.63) | 0.91 |
| smoking | Interaction | smoking:Vaccine/s adminsitered4 | 2.28 (0.21–18.34) | 0.46 |

**Table S1** This analysis is performed using Logistic regression on dataset of SHIP-START-3. Dependent variable is SARS-CoV-2 infection. Independent variables are type 2 diabetes Mellitus (T2DM), cancer and current smoking. Model was adjusted with number of administered vaccinations before infection, sex and age of the participants at the beginning of the study. Interaction between each independent variable (T2DM, Cancer and current smoking) and number of vaccinations was analyzed as well. OR (95%CI) stands for Odd Ratio and Confidence interval at 95%. In adjustment variables No vaccination and men are used as references for number of vaccination and sex respectively.

**Table S2** This analysis is performed using Logistic regression using SHIP-START-4 dataset. Dependent variable is SARS-CoV-2 infection. Independent variables are cancer and current smoking. Model was adjusted with number of administered vaccinations before infection, sex and age of the participants at the beginning of the study. Interaction between each independent variable (Cancer and current smoking) and number of vaccinations was analyzed as well. OR (95%CI) stands for Odd Ratio and Confidence interval at 95%. In adjustment variables No vaccination and men are used as references for number of vaccination and sex respectively.

| Independent Variable | Type | Term | OR(95%CI) | p_value |
| --- | --- | --- | --- | --- |
| **Cancer** | **Main Effect** | **Cancer** | **1.45 (0.86–2.46)** | **0.17** |
| Cancer | Main Effect | Vaccine/s adminsitered1 | 0.41 (0.11–1.49) | 0.17 |
| Cancer | Main Effect | Vaccine/s adminsitered2 | 0.53 (0.25–1.12) | 0.10 |
| Cancer | Main Effect | Vaccine/s adminsitered3 | 0.38 (0.21–0.65) | 0.00 |
| Cancer | Main Effect | Vaccine/s adminsitered4 | 0.13 (0.06–0.27) | 0.00 |
| Cancer | Main Effect | Sex (men reference) | 1.04 (0.75–1.44) | 0.81 |
| Cancer | Main Effect | Age | 0.98 (0.96–0.99) | 0.00 |
| **smoking** | **Main Effect** | **smoking** | **0.55 (0.34–0.88)** | **0.01** |
| smoking | Main Effect | Vaccine/s adminsitered1 | 0.42 (0.12–1.52) | 0.18 |
| smoking | Main Effect | Vaccine/s adminsitered2 | 0.53 (0.25–1.13) | 0.10 |
| smoking | Main Effect | Vaccine/s adminsitered3 | 0.38 (0.21–0.66) | 0.00 |
| smoking | Main Effect | Vaccine/s adminsitered4 | 0.13 (0.06–0.27) | 0.00 |
| smoking | Main Effect | Sex (men reference) | 1.06 (0.76–1.46) | 0.75 |
| smoking | Main Effect | Age | 0.98 (0.96–0.99) | 0.00 |
| Cancer | Interaction | Cancer:Vaccine/s adminsitered1 | 485417.37 (0–NA) | 0.98 |
| Cancer | Interaction | Cancer:Vaccine/s adminsitered2 | 0.73 (0.02–26.64) | 0.85 |
| Cancer | Interaction | Cancer:Vaccine/s adminsitered3 | 0.47 (0.02–3.38) | 0.51 |
| Cancer | Interaction | Cancer:Vaccine/s adminsitered4 | 1.37 (0.06–12.85) | 0.80 |
| smoking | Interaction | smoking:Vaccine/s adminsitered1 | 0 (NA–4.64653960382227e+35) | 0.98 |
| smoking | Interaction | smoking:Vaccine/s adminsitered2 | 1.06 (0.14–7.37) | 0.96 |
| smoking | Interaction | smoking:Vaccine/s adminsitered3 | 1.17 (0.25–4.98) | 0.84 |
| smoking | Interaction | smoking:Vaccine/s adminsitered4 | 3.37 (0.3–29.93) | 0.29 |
